# Tandem mass spectrometry of aqueous extract from *Ficus dubia* sap and its cell-based assessments for use as a skin antioxidant

**DOI:** 10.1038/s41598-021-96261-3

**Published:** 2021-08-19

**Authors:** Chaisak Chansriniyom, Rawiwan Nooin, Nitra Nuengchamnong, Ratjika Wongwanakul, Nalinrat Petpiroon, Wanwisa Srinuanchai, Bhanumas Chantarasuwan, Pornsiri Pitchakarn, Piya Temviriyanukul, Onanong Nuchuchua

**Affiliations:** 1 Department of Pharmacognosy and Pharmaceutical Botany, Faculty of Pharmaceutical Sciences, Chulalongkorn University, Bangkok, Thailand; 2 Natural Products and Nanoparticles Research Unit, Chulalongkorn University, Bangkok, Thailand; 3 Nano Agricultural Chemistry and Processing Research Team, National Nanotechnology Center (NANOTEC), National Science and Technology Development Agency (NSTDA), Pathumthani, Thailand; 4 Science Laboratory Center, Faculty of Science, Naresuan University, Phitsanulok, Thailand; 5 Nano Environmental and Health Safety Research Team, Advanced Nanocharacterization and Safety Research Group , National Nanotechnology Center (NANOTEC), National Science and Technology Development Agency (NSTDA), Pathumthani, Thailand; 6 National Science Museum, Technopolish, Klong 5, Klong Luang, Pathumthani, Thailand; 7 Department of Biochemistry, Faculty of Medicine, Chiang Mai University, Chiang Mai, Thailand; 8 Food and Nutrition Academic and Research Cluster, Institute of Nutrition, Mahidol University, Salaya, Phuttamonthon Nakhon Pathom, Thailand

**Keywords:** Metabolomics, Secondary metabolism

## Abstract

Since 2006, *Ficus dubia* has been reported as a new *Ficus* species in Thailand. As per our recent report, the red-brown aqueous extract of *F. dubia* sap (FDS) has been determined to strongly exhibit in vitro anti-radicals. However, the phytochemicals in the FDS extract related to health-promoting antioxidation have not been explored. Thus, in this study, we aimed to investigate the chemical components of the *F. dubia* sap extract by liquid chromatography-electrospray ionization quadrupole time-of-flight mass spectrometry (LC–ESI–MS/QTOF-MS) and its potential use in cosmetics in terms of cellular antioxidation on keratinocytes (HaCaT), phototoxicity, and irritation on 3D skin cell models following standard tests suggested by the Organization for Economic Cooperation and Development (OECD). It was found that the sap extract was composed of quinic acid and caffeoyl derivatives (e.g., syringoylquinic acid, 3-*O*-caffeoylquinic acid, 4-*O*-caffeoylquinic acid, and dimeric forms of caffeoylquinic acids). The extract has significantly exhibited antioxidant activity against H_2_O_2_-induced oxidative stress in HaCaT cells. The cellular antioxidative effect of the FDS extract was remarkably dependent on the presence of 3- and 4-*O*-caffeoylquinic acid in the extract. Furthermore, the FDS extract showed negative results on skin phototoxicity and irritation. Overall, the results reveal that the FDS extract could be developed as a new antioxidant candidate for a skin healthcare product.

## Introduction

Plants are an important source of phytochemicals that can be used for therapy and health promotion. Thus, discovery of new bioactive-rich extracts or active compounds can lead to the development of new medicines, active ingredients in dietary supplements, or cosmetic products. As per cosmetic purposes, extracts that maintain skin health properties and promote skin integrity are currently highly in demand in the world market.

The skin is the largest organ in the human body and serves as integumentary protection against harmful substances, such as ultraviolet (UV) radiation, oxidants, pollutants, and microbes. Excessive exposure to these substances often triggers stress on the skin. At levels of the outer living barrier, oxidative stress, which is an imbalance between reactive oxygen species (ROS) and anti-radical effectors, is consequential hostility and can cause various skin problems and even diseases^1^. For example, in the pathogenesis of psoriasis, intracellular ROS in keratinocytes adversely mediates inflammation through the mitogen-activated protein kinase signaling pathway^2^. Pollutants, such as airborne particulate matter, can generate excessive ROS and free radicals through chemical and biological processes, which, in turn, cause atopic dermatitis and acne vulgaris^3^. Moreover, ROS also play a vital role in skin aging by deteriorating the structural proteins like collagen and elastin with activated matrix metalloproteinases^4^. Therefore, the prevention of ROS generation or reduction or delay of ROS degenerative effect can be promising ways for skin protection.

Antioxidants are defined as substances counteracting oxidative radicals to terminate the redox chain reaction. Thus, antioxidant agents may help prevent skin damage and premature aging associated with oxidative stress. Ascorbic acid and tocopherols are effective antioxidants primarily used in skin care products^5^. Recently, natural antioxidants are being considered more economical, safe, and effective in treating or preventing ROS-induced skin problems^6^. Natural antioxidants from plant extracts are more abundant with phenolic compounds, such as phenolic acids, flavonoids, stilbenoids, ellagitannins, and phlorotannins. Quercetin and resveratrol, for instance, are in a group of flavonoids, which are well perceived by consumers^7^. The phenolic compounds can effectively scavenge and/or inhibit ROS production, resulting in reduced cellular ROS level^8^. Moreover, phenolic compounds have been shown to suppress intracellular inflammatory reactions^9,10^. Thus, in the cosmetic industry, finding a new natural antioxidant ingredient is a topic of high interest.

However, not much notice has been given to sap, a sticky exudate of tearing plant tissue. Among the sap-bearing plant families, Moraceae, especially the genus *Ficus*, is reported as a source of sap of medicinal importance for its anticancer^11,12^ and antihelminthic properties^13^ and inhibition of α-amylase and α-glucosidase^14^. One of the most interesting species is *Ficus dubia*, whose sap turns bloody red after exposure to air. This makes it a costly material like Dragon’s blood, which is a captivating crude drug characterized with red resin or sap obtained from *Croton* spp., *Daemonorops* spp., *Dracaena* spp., and *Pterocarpus* spp.^15,16^.

*Ficus dubia* is an indigenous plant distributed in the southern part of Thailand, Malaysia, Indonesia, and Brunei^17^. It is believed that the consumption of *F. dubia* sap would have health benefits. In fact, Suttisansanee et al.^18^ found that the extract of the *F. dubia* sap was rich in phenolics and flavonoids. The extract also promoted in vitro antioxidant activities determined by DPPH, ABTS, ferric reducing ability of plasma (FRAP), and oxygen radical absorbance capacity (ORAC) assays. Further, it was determined to also exhibit an antidiabetic mechanism via α-glucosidase inhibitory activity similar to the action of acarbose, which is a standard antidiabetic drug used to treat type 2 diabetes mellitus and prediabetes. Interestingly, both root and sap of the *F. dubia* were not genotoxic, as they did not present mutagenicity on a *Drosophila* wing-spot test. Antioxidant activities of the *F. dubia* sap seemed to be associated with its phenolic contents. However, only caffeic acid was found in the hydrolyzed sap. Thus, caffeic acid derivatives could be key components representing antioxidant activities in the *F. dubia* sap.

To develop the *F. dubia* sap as one of the cosmeceutical ingredients, in this work, we further investigated a chemical profile of the sap extract by using liquid chromatography-electrospray ionization quadrupole time-of-flight mass spectrometry (LC–ESI–MS/QTOF-MS); we further studied a cellular antioxidant activity of *F. dubia* sap extract on keratinocytes. Furthermore, phenolic acids in the extract were quantified and tested for antioxidant effects on keratinocytes. Additionally, we confirmed the safety of the extract using 3D skin models. We hope that this experiment can guide us to understand how *F. dubia* sap extract can be used as a skin antioxidant ingredient.

## Results

### Aqueous extract of *F. dubia* sap and its total phenolic content

In total, around 7.67% w/w of *F. dubia* sap (FDS) extract was obtained from the *F. dubia* sap. Its appearance was observed as a red-brown solid and freely soluble in water. The FDS extract contained a high number of phenolics, about 300.9 ± 9.6 mg gallic acid equivalence per gram extract (mg GAE/g extract).

### Phytochemical analysis of *F. dubia* sap extract using LC–ESI–MS/QTOF-MS

**Figure 1:**
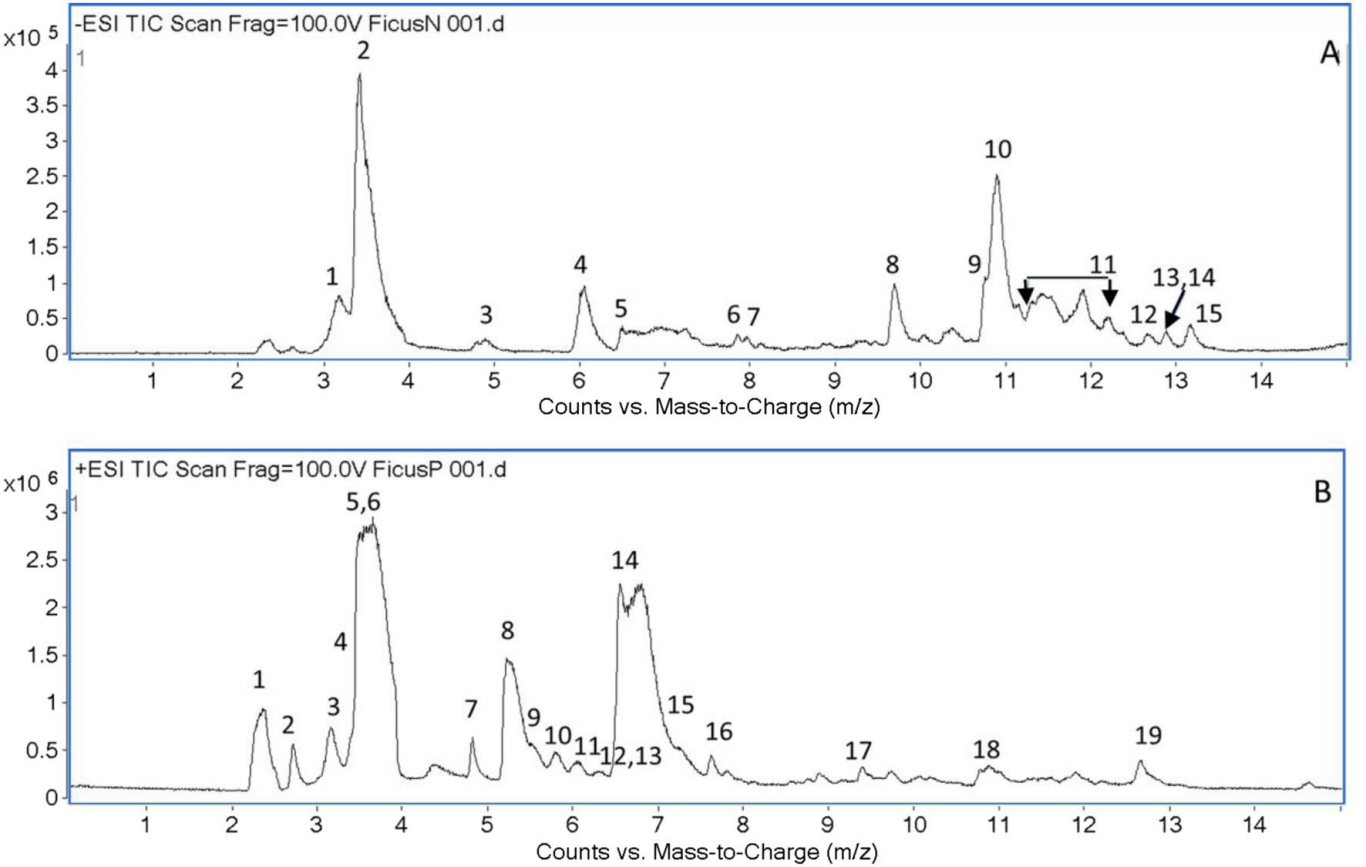
Total ion chromatogram of *F. dubia* sap extract at a concentration of 20 mg/mL operated in negative ionization mode (**A**) and positive ionization mode (**B**). The peak numbers were referred to the compounds listed in Tables 1 and 2.

The total ion chromatograms of FDS extract are presented in Fig. 1. Also, tentatively identified compounds with analytical parameters were listed in Tables 1 and 2. Three compounds (compounds **2**, **8**, and **10**) were initially identified by comparing retention time, accurate mass, and MS/MS fragments with authentic standards. Compound **2** with a molecular ion at *m/z* 191.0568 and retention time of 3.45 min was identified as quinic acid (Table 1). Additionally, the two isomers of *m/z* 353.08 at retention time of 9.7 and 10.9 min were identified as 3-*O*-caffeoylquinic acid (compound **8**, Table 1) and 4-*O*-caffeoylquinic acid (compound **10**, Table 1), respectively.

**Table 1:** Phytochemicals in *F. dubia* sap extract identified using LC–ESI–MS/QTOF method in the negative ionization mode.

No	RT (min)	*m/z* [M–H]^−^	MS/MS	Identification	Formula	Error (ppm)
1	3.16	179.0563	89.0226, 59.0126	hexose	C_6_H_12_O_6_	− 1.05
2	3.45	191.0568	191.0540, 173.0447, 127.0387, 85.0281, 59.0127	quinic acid	C_7_H_12_O_6_	− 3.6
3	4.91	191.0196	111.0070, 87.0058, 57.0344	citric acid	C_6_H_8_O_7_	0.66
4	6.05	186.0405	100.0388, 58.0284	1-(malonylamino)cyclopropanecarboxylic acid	C_7_H_9_NO_5_	1.59
5	6.66	163.0397	119.0488, 93.0327	4-hydroxycinnamic acid	C_9_H_8_O_3_	2.26
6	7.20	371.0982	353.0867, 191.0536, 135.0430	syringoylquinic acid	C_16_H_20_O_10_	0.46
7	7.96	371.0981	191.0541, 173.0430, 135.0436	syringoylquinic acid	C_16_H_20_O_10_	0.73
8	9.7	353.0888	191.0537, 179.0324, 135.0439, 85.0281	3-*O*-caffeoylquinic acid	C_16_H_18_O_9_	-2.82
9	10.74	705.1682	513.1015, 353.0853, 339.0488, 191.0543, 173.0437	caffeoylquinic acid dimer	C_32_H_34_O_18_	− 1.36
10	10.90	353.0894	191.0540, 173.0436, 135.0433, 93.0336, 85.0286, 59.0126	4-*O*-caffeoylquinic acid	C_16_H_18_O_9_	− 4.52
11	11.88	705.1697	513.1015, 339.0497, 191.0543, 173.0437	caffeoylquinic acid dimer	C_32_H_34_O_18_	− 3.49
12	12.66	270.0763	213.0484, 137.0213, 119.0489, 93.0318	acetaminosalol	C_15_H_13_NO_4_	3.25
13	12.88	206.0814	164.0707, 147.0436, 58.0281	2-acetamido-4-methylphenyl acetate	C_11_H_13_NO_3_	4.21
14	12.88	250.0713	164.0703, 147.0416, 103.0541, 70.0298, 58.0273	*N*-carboxyacetyl-d-phenylalanine	C_12_H_13_NO_5_	3.18
15	13.17	121.0284	92.0253, 65.0384	2-hydroxybenzaldehyde	C_7_H_6_O_2_	10.68

**Table 2:** Phytochemicals in *F. dubia* sap extract identified using LC–ESI–MS/QTOF method in the positive ionization mode.

No	RT (min)	*m/z* [M+H]^+^	MS/MS	Identification	Formula	Error (ppm)
1	2.32	185.0674	127.0241, 83.0345, 68.9826	pentane-heptol	C_5_H_12_O_7_	− 9.84
2	2.70	104.1078	60.0813	choline	C_5_H_14_NO	− 2.51
3	3.14	136.0766	119.0494, 91.0546, 77.0390, 65.0390	2-phenylacetamide	C_8_H_9_NO	− 6.68
4	3.36	330.1568	312.1447, 222.1143, 150.0918, 91.0546	2,3,4,5,6,7-hexahydroxy-*N*-(2-phenylethyl) heptanamide	C_15_H_23_NO_7_	− 6.27
5	3.59	168.1031	150.0918, 135.0682, 107.0496, 91.0547, 77.0389	synephrine	C_9_H_13_NO_2_	− 7.11
6	3.59	150.092	135.0682, 107.0493, 91.0546, 77.0389	*N*-benzylacetamide	C_9_H_11_NO	− 4.39
7	4.84	241.155	196.0935, 128.1063, 84.0812, 58.0653	ethyl(3-oxodecahydro-2-quinoxalinyl) acetate	C_12_H_20_N_2_O_3_	− 1.37
8	5.24	152.1076	121.0651, 91.0548, 77.0391	*N*-methyltyramine	C_9_H_13_NO	− 4.01
9	5.52	180.1392	121.0651, 77.0389, 60.0812	3-(dimethylamino)-1-phenyl-1-propanol	C_11_H_17_NO	− 5.05
10	5.77	166.1236	150.0916, 121.0652, 91.0548, 77.0389	2-(3-methoxyphenyl)-1-propanamine	C_10_H_15_NO	− 5.78
11	6.11	170.0456	124.0394, 96.0449, 66.0343	2-furoylglycine	C_7_H_7_NO_4_	− 4.8
12	6.35	194.1184	179.0945, 164.0707, 136.0760, 108.0813, 93.0575	butyl 2-aminobenzoate	C_11_H_15_NO_2_	− 4.35
13	6.37	210.1134	164.1069, 149.0835, 123.0443, 95.0495, 77.0392, 58.0652	ethyl l-tyrosinate	C_11_H_15_NO_3_	− 4.43
14	6.76	670.3732		methylenemescaline trimer	C_36_H_51_N_3_O_9_	− 5.06
	6.77	447.2509	224.1285, 165.0549, 123.0441, 95.0495, 60.0815	1,4-bis(2,3,4-trimethoxybenzyl)piperazine	C_24_H_34_N_2_O_6_	− 4.33
15	7.34	330.1349	190.0862, 150.0914, 123.0446, 91.0545	(*R*)-*N*-trans-feruloyloctopamine	C_18_H_19_NO_5_	− 3.94
16	8.61	330.1343	270.1126, 107.0496	1-*O*-acetyllycorine	C_18_H_19_NO_5_	− 2.12
17	9.44	358.1668	252.1233, 209.0816, 137.0593, 107.0492, 58.0656	3-[4-({2-[(3-methoxybenzyl)oxy]propanoyl}amino)phenyl]propanoic acid	C_20_H_23_NO_5_	− 5.31
18	10.85	510.2125	331.1395, 277.1071, 185.0811	unidentified		
19	12.69	272.0924	226.0865, 166.0499, 165.0425, 137.0474, 107.0500, 82.0293	acetaminosalol	C_15_H_13_NO_4_	− 2.45

**Figure 2:**
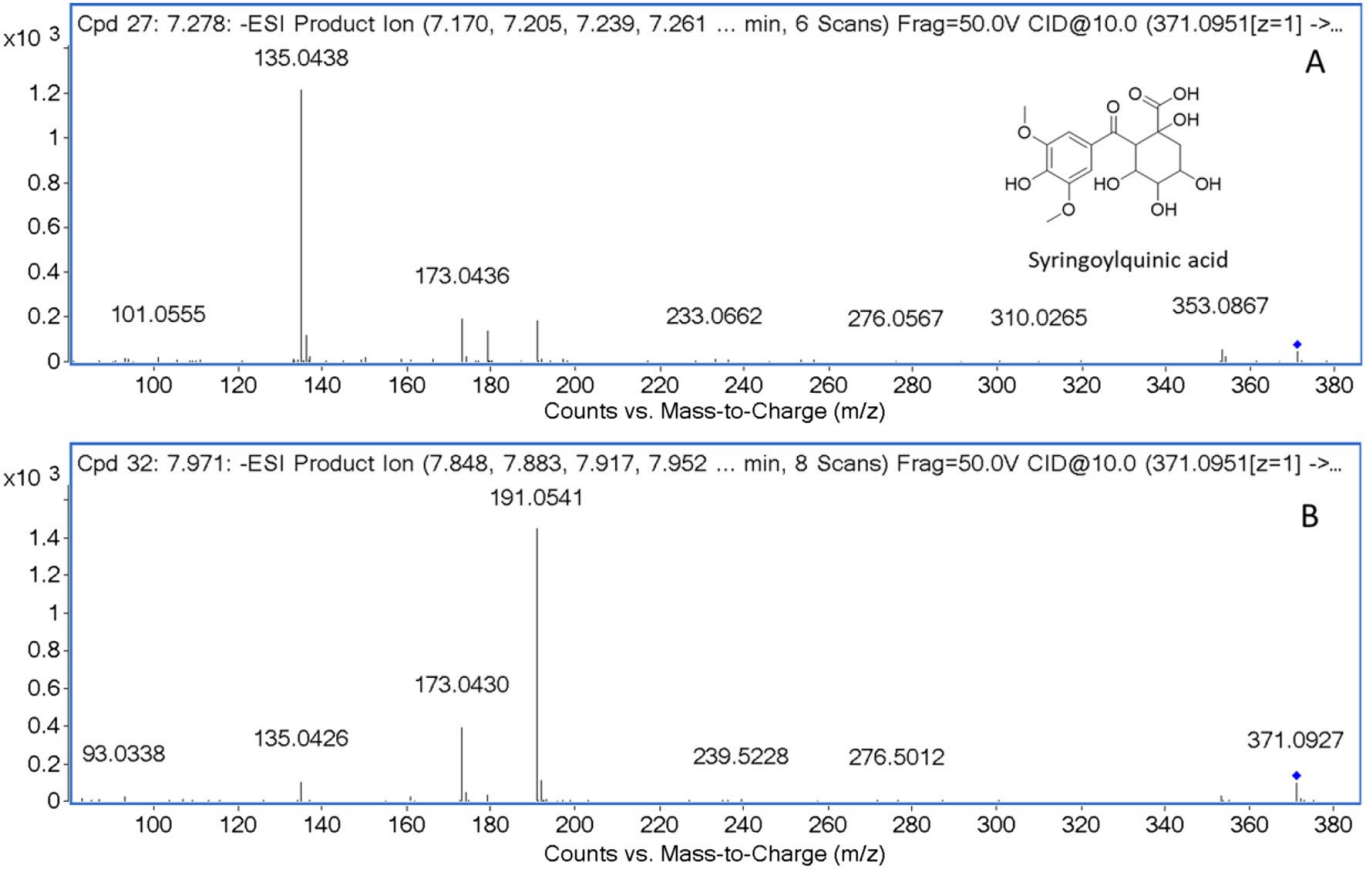
MS/MS data and proposed structure of syringoylquinic acid, compound **6** (**A**), and **7** (**B**).

Two isobaric compounds **6** and **7** were noted to share the same deprotonated molecule [M−H]^−^ at *m/z* 371.098 (Table 1). In ( −)-ESI/MS/MS, compound **7** showed a base peak at *m/z* 191.055 [quinic acid–H]^−^, corresponding to the loss of syringoyl moiety. Compound **6** produced a base peak at *m/z* 135.04 [syringoyl–CO–OH]^−^. These two compounds were tentatively identified as syringoylquinic acid, but the conjugation position could not be clarified. The fragmentation patterns are shown in Fig. 2.

**Figure 3:**
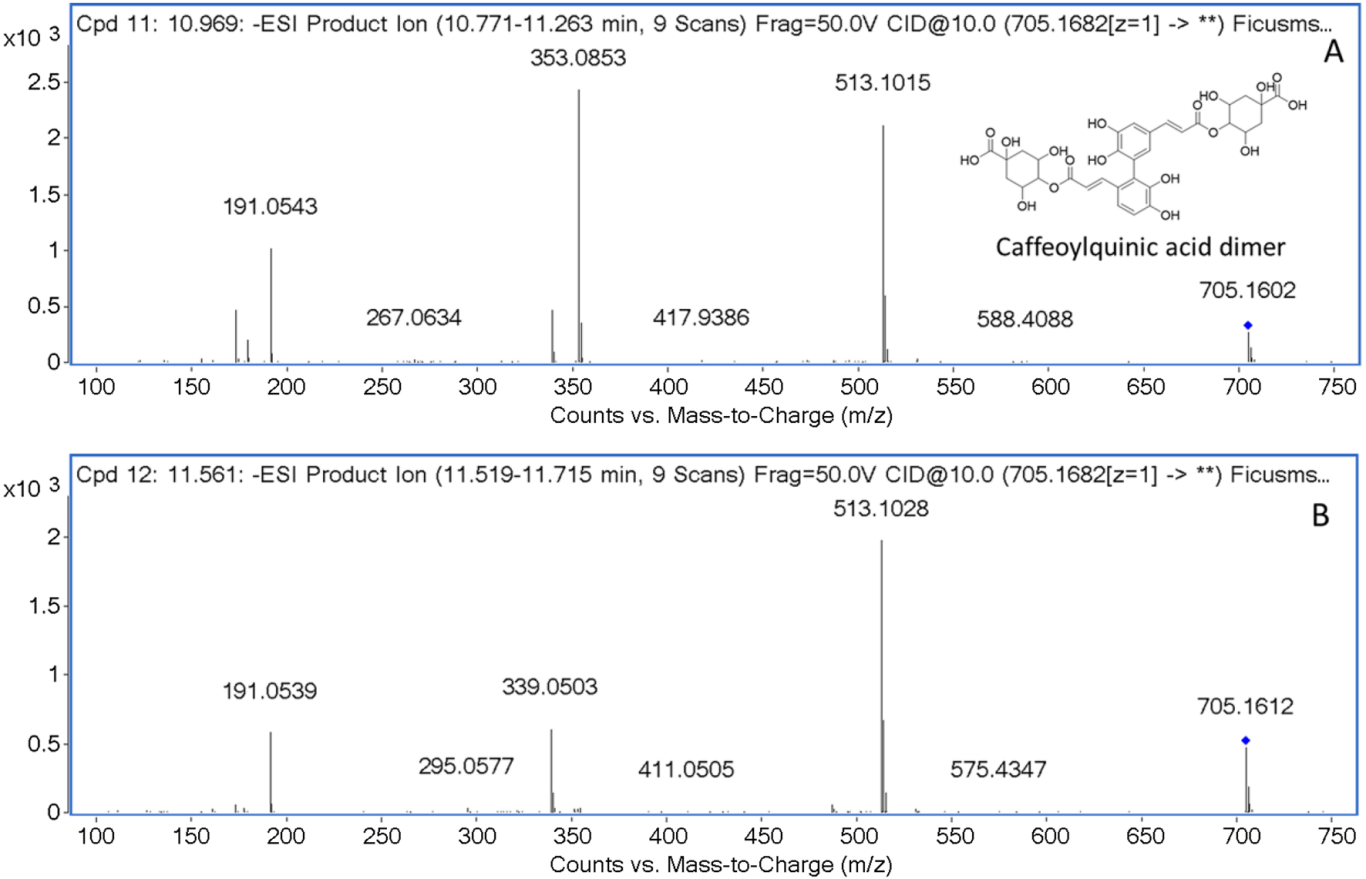
MS/MS data and proposed structure of caffeoylquinic acid dimer and compounds **9** (**A**) and **11** (**B**).

Compounds **9** and **11** showed the same molecular ion at *m/z* 705.169 and fragment ions at *m/z* 513 [M–192]^−^, 353.08 [caffeoylquinic acid]^−^, 191.05 [quinic acid]^−^, and 173 [quinic acid–H_2_O]^−^. The characteristic of 353, 191, and 173 implied that it was 4-*O*-caffeoylquinic acid. Then, these two compounds were proposed as caffeoylquinic acid dimers. Nonetheless, the position was not confirmed. The MS/MS data were shown in Fig. 3.

In positive ionization mode, the molecular ion mostly appeared as an even number, implying that there was at least one nitrogen compound in their molecules. According to the MS/MS spectra, *m/z* at 91 [methylbenzyl]^+^, 77 [benzyl]^+^ were of compounds **3**–**6** and **8**–**10**. These compounds were proposed as 2-phenylacetamide (**3**), 2,3,4,5,6,7-hexahydroxy-*N*-(2-phenylethyl)heptanamide (**4**), synephrine (**5**), *N*-benzylacetamide (**6**), *N*-methyltyramine (**8**), 3-(dimethylamino)-1-phenyl-1-propanol (**9**), and hordenine (**10**). The other amines are listed in Table 2.

The MS fragmentation and proposed structure of all compounds in the FDS extract were shown in Supplementary Data (Figures S1 and S2).

### Quantification of quinic acid and 3- and 4-caffeoylquinic acids in *F. dubia* sap extract

**Figure 4:**
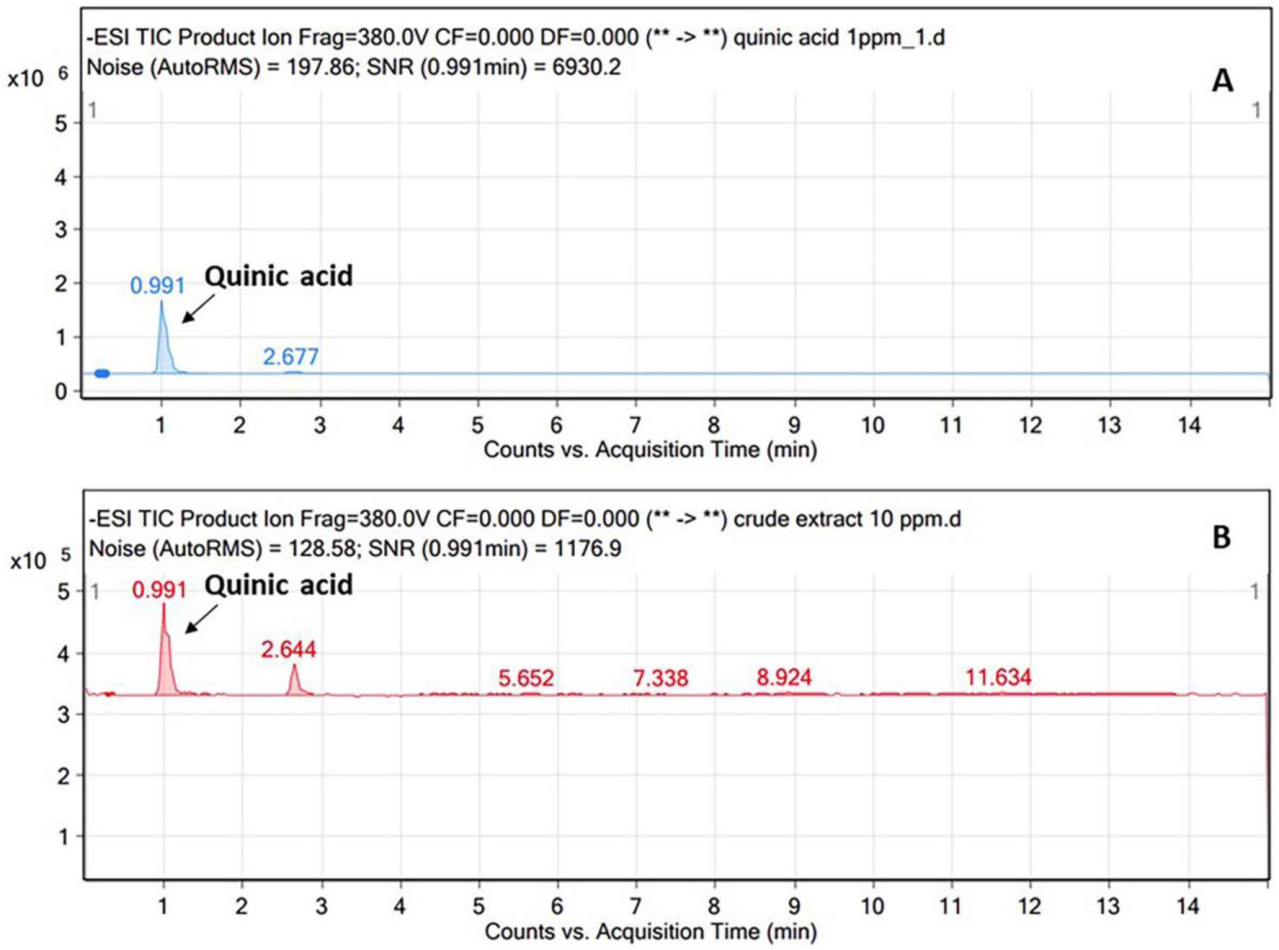
LC–MS chromatograms of (**A**) standard quinic acid and (**B**) quinic acid in *F. dubia* sap (FDS) extract.

**Figure 5:**
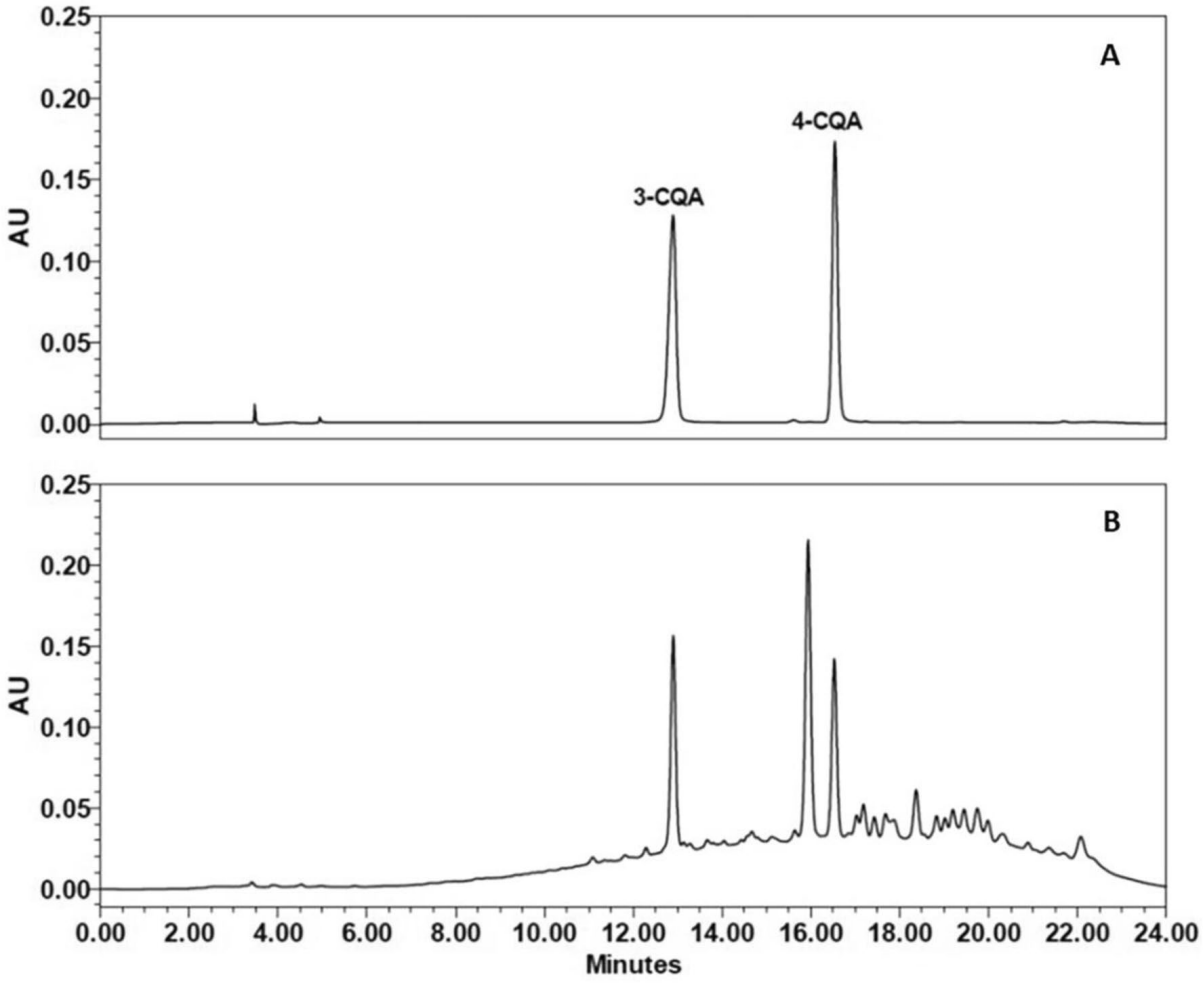
HPLC–DAD chromatograms of (**A**) mixed chlorogenic acid standards of 3-*O*-caffeoylquinic acid (3-CQA; *t*_r_ = 12.883 min) and 4-*O*-caffeoylquinic acid (4-CQA; *t*_r_ = 16.509 min), at a concentration of 50 ppm, and (**B**) *F. dubia* sap (FDS) extract (10 mg/mL). The peaks were detected at a wavelength of 330 nm.

Quinic acid was quantified using LC–ESI–MS (Fig. 4), while 3- and 5-*O*-caffeoylquinic acids were determined using the HPLC method (Fig. 5). The validation parameters are presented in Table S1 (see Supplementary Material). It was found that the FDS extract in this study contained 1.32 ± 0.15% w/w quinic acid (QA), 0.39 ± 0.01% w/w 3-*O*-caffeoylquinic acid (3-CQA), and 0.34 ± 0.00% w/w 4-*O*-caffeoylquinic acid (4-CQA). Unfortunately, this study has not yet quantified other caffeoylquinic derivatives such as syringoylquinic acid (compounds **6** and **7**) and dimeric forms of caffeoylquinic acid (compounds **9** and **11**). Therefore, isolation technique is needed to further separate those compounds.

The concentrations of QA, 3-CQA, and 4-CQA presented in the FDS extract were used to study the cellular antioxidation in keratinocytes in the next experiment.

### Cellular antioxidation activities of *F. dubia* sap extract

**Figure 6:**
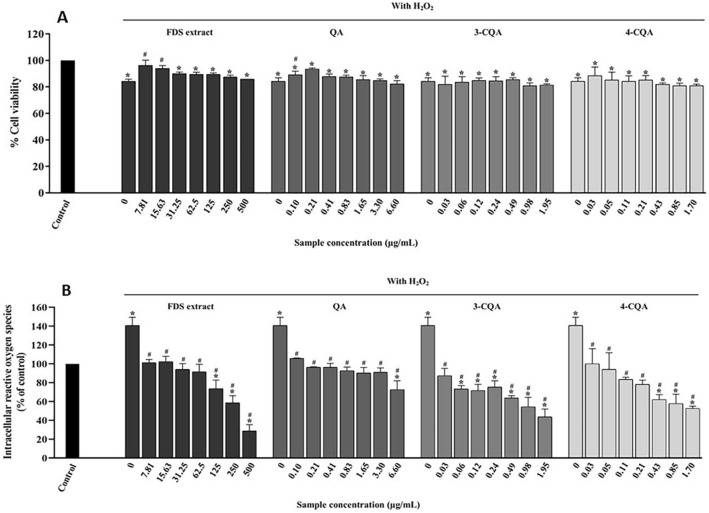
(**A**) HaCaT cell viability was determined via MTS assay. The cells were treated with various concentrations of the *F. dubia* sap (FDS) extract, quinic acid (QA), 3-*O*-caffeoylquinic acid (3-CQA), or 4-*O*-caffeoylquinic acid (4-CQA) for 24 h before exposure to H_2_O_2_ (100 µM) for 30 min. (**B**) Inhibition of H_2_O_2_-induced ROS generation by the extracts and compounds in HaCaT cells. The cells were pretreated with the FDS extract, QA, 3-CQA, or 4-CQA for 24 h and then treated with H_2_O_2_ for 30 min. The levels of intracellular reactive oxygen species were monitored after an inner salt 2′,7′dihydrofluorescein diacetate fluorescent dye was introduced into the cells. The symbols * and # represent significantly different data at *p*-values ≤ 0.05 compared to non-H_2_O_2_-treated cells (control) and H_2_O_2_-induced oxidative stress cells without any tested compounds (0 µg/mL). The experiments were conducted in triplicate.

Cellular antioxidant activities of the FDS extract and their identified components were conducted in HaCaT cells. Cell viability and intracellular reactive oxygen species (intracellular ROS) were assessed to determine the cytoprotective capability of the FDS extract against H_2_O_2_-induced oxidative stress. The H_2_O_2_ concentration for inducing oxidative stress was selected at 100 µM, which exhibited 84.42 ± 2.31% of survival cells compared with a control group (untreated with H_2_O_2_) (Fig. 6A at 0 µg/mL of sample solution). The FDS extract at 0–500 µg/mL, QA, and chlorogenic acids at equivalent concentrations presented in the FDS extract significantly restored cell viability upon oxidative stress (Fig. 6A). Moreover, they rather scavenged the ROS in keratinocytes, as shown in Fig. 6B. The extract at concentrations of 7.81 and 15.83 μg/mL has significantly decreased intracellular ROS to a level similar to the control group. An increase in the extract concentration up to 500 μg/mL further dramatically decreased the intracellular ROS.

The cytoprotective effects of chlorogenic acid derivatives were also observed at concentrations existing in the extract at 0–500 µg/mL. Based on chemical quantification, the derivatives were observed in ranges of 0–6.60 μg/mL QA, 0–1.95 μg/mL 3-CQA, and 0–1.70 μg/mL 4-CQA, respectively. The ROS scavenging activity of the FDS extract was likely related to its phytochemical compositions, such as 3-CQA and 4-CQA, rather than QA. The cytoprotective dose of the extract was 125–500 µg/mL, which contained about 0.49–1.95 µg/mL of 3-CQA and 0.43–1.70 µg/mL of 4-CQA. At these concentrations, 3-CQA and 4-CQA were noted to have significantly exhibited the depleting effect on intracellular ROS without toxicity on keratinocytes. Among the test compounds, the protective effect was marked on 3-CQA at 1.95 µg/mL, which exhibited the highest decrease in intracellular ROS to 43.49 ± 8.61%. Accompanied by 3-CQA and 4-CQA, our results suggest that 3-CQA and 4-CQA can influence the intracellular decreasing manner of the extract in higher concentrations.

### 3T3 neutral red uptake phototoxicity test of *F. dubia* sap extract

The phototoxicity potential of the FDS extract using the 3T3 neutral red uptake test is presented in Table 3. The half-maximal inhibitory concentration (IC_50_) of chlorpromazine hydrochloride (CPZ) upon non-UV- and UV-irradiated conditions was accepted, regarding the recommendation of the OECD TG 432 (see Table 3). The IC_50_ of CPZ should be in ranges of 7.0–90.0 μg/mL without UV irradiation and 0.1–2.0 μg/mL after UV-irradiated condition. The mean photo effect (MPE) and photo-irritation-factor (PIF) values of CPZ also showed phototoxicity. It suggested that the experimental procedure used in this study was validated.

**Table 3:** The half-maximal inhibitory concentration (IC_50_), mean photo effect (MPE), and photo-irritation-factor (PIF) values of chlorpromazine hydrochloride (CPZ) and the *F. dubia* sap (FDS) extract from the 3T3 neutral red uptake phototoxicity test.

Sample	IC_50_ (µg/ml)		MPE	PIF	Prediction
	−Irr	+Irr			
CPZ	27.46 ± 1.32	1.05 ± 0.23	0.56 ± 0.08	26.87 ± 4.26	Phototoxicity
FDS extract	> 1000	> 1000	0.02 ± 0.01	–	No phototoxicity

MPE and PIF data represent the mean ± SD of three independent assays.

The experiments were conducted in triplicate.

−Irr; non-UV irradiated conditions and +Irr; UV irradiated conditions.

The phototoxicity analysis of the FDS extract showed no cytotoxicity whether the cells were irradiated or non-irradiated by UV. Furthermore, the IC_50_ of the FDS extract was observed to be above 1000 µg/mL. Thus, the IC_50_ was unable to determine the phototoxicity of the FDS extract. In this case, the MPE value can be used instead for predicting the phototoxicity of the FDS extract. In this study, the MPE value of the FDS extract was 0.02 ± 0.01 (see Table 3). Thus, it can be considered that the FDS extract has no phototoxicity.

### Skin irritation test of the *F. dubia* sap extract

**Figure 7:**
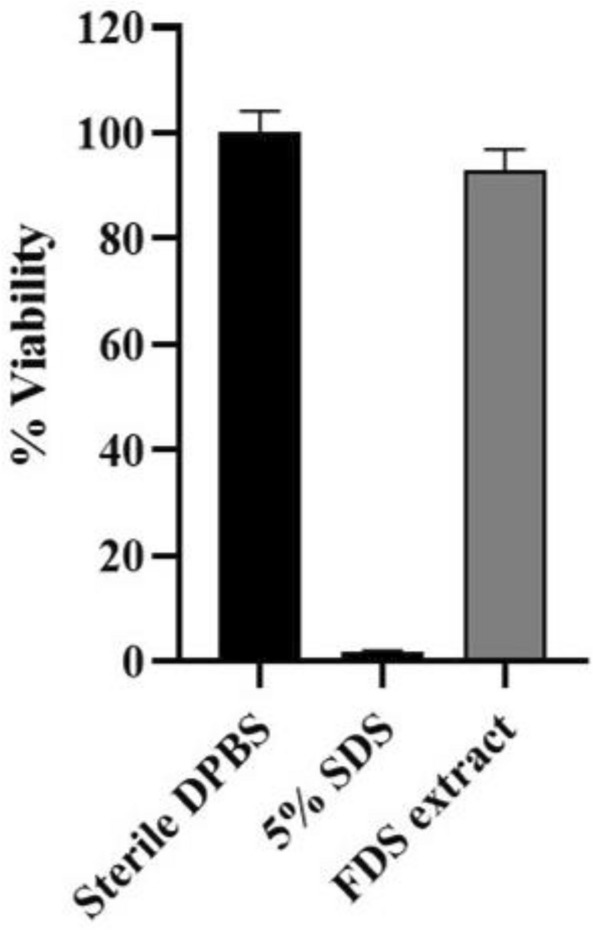
%Viability of the 3D skin model (EpiDerm) was measured after treating with sterile Dulbecco’s phosphate-buffered saline (DPBS) as a negative control, 5% sodium dodecyl sulfate (SDS) as a positive control, and the *F. dubia* sap (FDS) extract at 830 mg/mL. The experiments were conducted in triplicate.

The skin irritation assay can be measured by the percentage of human epidermis tissue (EpiDerm). The 3D skin model remained 100.00 ± 4.20% viability when the tissue was treated with a buffer. In comparison, the tissue showed irritation effect by a dramatic decrease in cell viability to nearly 0 after 5%sodium dodecyl sulfate (SDS) was introduced. Interestingly, the FDS extract at a concentration of 830 mg/mL did not cause skin irritation as the tissue viability was 92.94 ± 4.03% (Fig. 7).

## Discussion

*Ficus dubia* is a *Ficus* plant distributed in a limited area in the southern forest of Thailand. A study conducted by Suttisansanee et al.^18^ was the first published work examining the antioxidant, antidiabetic, anti-Alzheimer’s, and anticancer benefits of *F. dubia* root and sap extracts. Moreover, the extracts had no genotoxic effects observed by *Drosophila* wing-spot test. Interestingly, the *F. dubia* sap (FDS) presented effective antioxidant activities indicated by ORAC (about 8000 µmol Trolox equivalents per gram extract). Thus, this study exploits the sap as an antioxidant substance for the skin and unveils its phytochemical and antioxidative components via LC–ESI–MS/QTOF-MS. The sap material used in this research was collected from the exact location where Suttisansanee et al. sourced their *F. dubia* plants^18^. The number of total phenolics controlled the quality of the FDS. In this study, total phenolic content (TPC) found in the FDS extract was closed as previously reported^18^.

The *F. dubia* sap was determined to be abundant with phenolic compounds as commonly found in *Ficus* species and reported as major phytochemicals contributing to antioxidant activities. Most studies on TPC and antioxidant activities were reported on the fruit and latex of *F. carica* (common fig)^19–22^. The TPCs of figs were determined, depending on cultivars and extraction methods^22^. In cases of aqueous and alcoholic extractions, the skin of fresh fig, cultivar “VB1,” contained the total phenolic content of about 140 mg/100 g fresh weight^20^. The “White Genoa” *F. carica* cultivar showed about 300 μg GAE/mL. It revealed that the sap of *Ficus* is a good source of phenolics. The high content of phenolics found in FDS extract was further investigated for its identified components.

The phenolic compounds in the FDS extract were determined in our study. It mainly comprised of QA and its acyl derivatives, that is, chlorogenic acids. Such a high amount of QA found in the extract suggested the phenolic derivatives are from the shikimate pathway. It is also possible that QA could be a metabolite used for biosynthesis of chlorogenic acids. QA was found in the leaves of Portuguese *F. carica* varieties “Pingo de mel” and “Branca Tradicional”, containing 10,502.5 ± 97.1 mg/kg and 5183.3 ± 116.5 mg/kg of lyophilized extracts, respectively. In addition, citric acid (*m/z* 191.0196) was found in the FDS extract, a similar component in *F. carica* latex^23^. Moreover, 3- and 4-*O*-caffeoylquinic acid were identified in the FDS extract by observing the [M–H]^−^ at *m/z* of 353.0888 and 353.0894, respectively. Further comparison of the MS/MS pattern and HPLC chromatogram with authentic compounds was able to confirm their caffeoyl position. In addition, it is also supported by the existence of caffeic acid in the acid-hydrolyzed *F. dubia* sap^18^. 3-*O*-Caffeoylquinic acid (3-CQA) was identified by the base peak at *m/z* of 191.0537 and secondary peak at 179.0324 m*/z*, while 4-CQA was confirmed by the prominent base peak at *m/z* of 173.0436. 3-*O*-Caffeoylquinic acid was also detected in small amounts in the peel of “Pingo de mel”, while 4-*O*-caffeoylquinic acid was also found in nine Tunisian dry fig cultivars in the range of 0.10 to 2.42 mg/100 g of dry fig^21^. Steroidal and triterpenoid compounds found in pet ether extract of *F. carica* latex exhibited cytotoxic activities toward cancer cell lines^11,24^. Ghanbari et al.^11^ have also mentioned the phenolic substances presented in the latex; however, they were not exactly identified. Two research works by Oliveira et al.^23,25^ revealed the latex metabolites of *F. carica*, which are phytosterols (especially *β*-sitosterol), terpenoids, fatty acids, amino acids, and organic acids. It was reported on an antioxidant against DPPH^·^ at IC_25_ of 1049 μg/mL. Additionally, it is interesting to note that QA and its acyl derivatives (chlorogenic acids) in *Ficus dubia* sap were found in high amounts as similar to those in *Lecokia cretica* and *Inula graveolens* extracts, which are known for their antioxidant activities^26,27^.

The epidermis is the outer skin layer. Keratinocytes, which are the major cells in the epidermis, are often deteriorated by oxidants resulting from physiological processes and harmful stimuli, such as UV radiation and particulate matter pollution^4^. Thus, keratinocytes are a target for skin delivery of antioxidants to nourish and maintain their integrity. Several natural compounds, such as dendropachol^28^ and ellagic acid^29^, are known to exhibit protective effects against oxidative stress. This study found that the FDS extract demonstrated a dose-dependent manner in decreasing intracellular ROS and maintained almost 90% cell survival compared to the control. The FDS extract at concentrations of 7.80 and 15.63 μg/mL recovered the cell viability (up to 95%) and decreased the intracellular ROS to the normal condition (nearly 100%). The ROS reduction and cell survival patterns of FDS extract reflected the cell protective tendency of the extract influenced by QA, 3-CQA, and 4-CQA. Compared to 3-CQA, QA exhibited weak antioxidant activities on in vitro antioxidant assays underlying mechanism of single electron transfer (SET) such as Trolox equivalent antioxidant capacity (TEAC) and FRAP because of its lack of extended π-system to stabilize the radical cation^30^. Results demonstrated that 3-CQA and 4-CQA were superior to QA in terms of reducing intracellular ROS (*p* < 0.05). In concordance with the results studied by Gul et al.^31^, chlorogenic acid at a concentration of 0.11 ± 0.01 μM exhibited 50% inhibition of ROS induced by H_2_O_2_ in rat cortical slices. Moreover, many studies also claimed the protective effect of chlorogenic acid on cells such as osteoblast (MC3T3-E1) and lens epithelial (HLE-B3) cells from H_2_O_2_-induced oxidative stress^32,33^.

Evaluation of FDS extract on skin toxicity is necessary before using it as an active ingredient in antioxidant skin products. There are some case reports on phytophotodermatitis after getting in contact with *Ficus* sap. This skin toxicity can be attributed to the presence of photoactive furocoumarins (e.g., psoralen and bergapten) in the *Ficus* sap^34,35^. However, results demonstrated that FDS extract did not show phototoxicity and skin irritation regarding the 3T3-neutral red uptake and 3D skin tests, respectively. Our extraction process using DI water as an extraction solvent may contribute to the nontoxic properties of the extract. Moreover, the mass adducted of possible furocoumarins were not found in the extract analyzed via LC–ESI–MS/QTOF-MS. Thus, these furocoumarins, which are hydrophobic, were excluded from the extract. Hence, FDS extract was able to pass the preliminary tests, confirming its safety to the skin. Alternatively, amine compounds were found in FDS (Table 2). *N*-Methyltyramine (**8**) and hordenine (**10**), which are biogenic amines found in various plants, were reported as toxic compounds on animals^36^, but not specifically related to human skin.

Chlorogenic acids demonstrate direct antioxidant activity in human diets; these days, they are also used for skin protection in skincare and cosmetic products. Coffee beans are known as a source of chlorogenic acids comprising of 5-CQA as the main component^37^. In this paper, we suggest FDS extract as a new source of chlorogenic acid derivatives, which is enriched with 3- and 4-COA. Based on our results, the FDS extract could be a promising new antioxidant use in cosmetics.

## Material and methods

### Chemicals and reagents

Dulbecco’s Modified Eagle Medium (DMEM) containing phenol red, penicillin–streptomycin, EDTA-trypsin, and Dulbecco’s phosphate buffer solution (DPBS) was purchased from Gibco BRL Life Technologies (Invitrogen™, Paisley, UK). Fetal bovine serum (FBS) was purchased from HyClone, USA. 3-(4,5-Dimethylthiazol-2-yl)-5-(3-carboxymethoxyphenyl)-2-(4-sulfophenyl)-2H-tetrazolium, inner salt (MTS) was purchased from Promega, USA. Hydrogen peroxide, 2′,7′dihydrofluorescein diacetate (DCFH-DA), chlorpromazine hydrochloride (CPZ), QA, neochlorogenic acid (CAS No. 906-33-2) with IUPAC name 3-*O*-caffeoylquinic acid (3-CQA), and cryptochlorogenic acid (CAS No. 905-99-2) with IUPAC name 4-*O*-caffeoylquinic acid (4-CQA) were purchased from Sigma-Aldrich, United Kingdom. Acetonitrile (LC–MS reagent) was purchased from RCI Labscan (Bangkok, Thailand). Water type I was purified from MilliQ^®^ Integral 3 system (Millipore, Bedford, MA, USA). Formic acid (analytical grade) was purchased from Merck (Darmstadt, Germany). A 0.45 mm nylon disposable membrane filter and syringe filter were supplied from Vertical Chromatography, Thailand. Other unmarked reagents were of analytical grade.

### Preparation of aqueous extract of *F. dubia* sap

It is noted that all methods including a sample collection were performed in accordance with the relevant guidelines and regulations. Briefly, *F. dubia* sap (FDS) was collected by Dr. Bhanumas Chantarasuwan in January 2019 from Hala-Bala Wildlife Sanctuary, Waeng District, Narathiwat, Thailand. The sample collection was conducted following the guidelines and regulations of the legislation number 0907.4/25755, as authorized by the Department of National Parks, Wildlife and Plant Conservation, Chatuchak, Bangkok, Thailand. The *F. dubia* specimen (voucher number: Chantarasuwan 040117-1) was authenticated and deposited at the Natural History Museum of Thailand, Pathum Thani, Thailand.

Briefly, the *F. dubia* sap is harvested by slicing 15–20 cm and 15°–25° angle of a groove into the tree’s cambium with a hooked knife. The grooves were made until 1 L of the sap was collected. The sap (1000 g) was lyophilized for 48 h. Then, the dried sample was re-dissolved with deionized water to remove resinous and other water-insoluble substances. The solution was slowly stirred by a magnetic stirrer for an hour and then filtered through Whatman filter paper No. 1. The red-brown filtrate was then collected and lyophilized for 48 h. Then, the aqueous FDS extract powder (76.7 g) was obtained and kept in an aluminum foil bag and stored at − 20 °C for further studies.

### Phytochemical analysis

#### Determination of TPC in the *F. dubia* sap extract by spectrophotometer

The TPC of the FDS extract was determined using the Folin-Ciocalteu method^38^. The extract (20 µL) at different concentrations (12.5, 25, 50, 100, 200, and 400 µg/mL) was mixed with 100 µL of 10% Folin–Ciocalteu solution and 80 µL of 7.5% sodium bicarbonate solution in a 96-well plate. The mixture was gently mixed and stored in the dark for 30 min. Later, the absorbance of mixture solutions was measured at 765 nm by spectrophotometer (SpectraMax, Molecular Devices, USA).Total phenolic contents were calculated using the calibration curve of gallic acid. The values were expressed as mg gallic acid equivalent per gram of extract (mg GAE/g extract).

#### Phytochemical screening by liquid chromatography-electrospray ionization quadrupole time-of-flight mass spectrometry (LC–ESI–MS/QTOF-MS)

A protocol of LC–ESI–MS/QTOF-MS was modified from research works of Nuengchamnong et al.^39^ and Duangjai et al.^40^. Briefly, the LC–ESI–MS/QTOF-MS system consists of HPLC unit 1260 infinity series (Agilent Technologies, Waldbronn, Germany) coupled with an ultra-high-definition accurate mass spectrometer (Agilent Technologies, Singapore). Chromatographic separation of the FDS extract (concentration 20 mg/mL of water) was conducted on a Luna C18(2) column (150 × 4.6 mm, 5 μm, Phenomenex, USA) at a flow rate of 0.5 mL/min. The column temperature was maintained at 35 °C. The mobile phase consisted of A (0.1% formic acid in type I water, v/v) and B (0.1% formic acid in acetonitrile, v/v). The elution gradient was started from 5 to 20% B for 5 min, then changed to 50% B in 10 min, and increased to 80% B in 2 min, and held for 3 min. The total run was 15 min, and the post-run was for 5 min. The injection volume was 10 mL using an autosampler. The dual electrospray ionization (ESI) source was operated in both negative and positive modes. The ESI condition was as follows: drying gas (gas N_2_) temperature, 350 °C; gas flow rate, 10 L/min; nebulizer pressure, 30 psig; mass range, 100–1000 m*/z*; scan rate, 4 spectra/s; capillary voltage, 3500 V; skimmer voltage, 65 V; octapole RFV, 750 V; and fragment voltage, 50 V. The automatic fragmentation pattern was set with collision energy at 10, 20, and 40 V using UHP N_2_ gas. Accurate mass measurements (error < 5 ppm for analytes) were obtained through an automated calibrant delivery system on a daily basis using a dual-nebulizer ESI source (calibrant solution B, Agilent Technologies, USA). Two reference masses were constantly introduced during the acquisition and used for drift correction (calibrant solution A, Agilent Technologies, USA). All acquisition and analysis of the data used MassHunter Data Acquisition Software B.05.01 and MassHunter Qualitative Analysis Software B 06.0 (Agilent Technologies, USA). To identify the compounds, peak retention time, mass data, and their fragmented ions were compared to those of registered compounds on public databases: Human Metabolome Database, lipid maps, METLIN Metabolomics Database and Library (Agilent Technologies), and authentic compound. The mass error was calculated when comparing a theoretical *m/z* and an experimentally observed *m/z* of an assignment.

#### Quantification of QA and its derivatives

A quantitative analysis of QA was conducted using an Agilent LC–MS system (Agilent 1290 Infinity Series, Waldbronn, Germany). The chromatogram of QA was conducted using Agilent Poroshell 120 (2.1 mm × 100 mm, 2.7 μm). The mobile phase consisted of (A) deionized water and (B) acetonitrile: 5-mM phosphate buffer (80:20). The flow rate of the mobile phase was kept at 0.5 mL/min. The gradient elution starting from the low content of A was as follows: 0–7 min, gradient mixture from 0% A:100% B to 6% A:94% B; 7–10 min, and the gradient mixture 6% A:94% B to 0% A:100% B. Later, it was held at 0% A for 5 min. The injection volume was 5 µL. The mass spectrometric analysis was performed using an Agilent 6495 Triple Quadrupole operating in negative ionization mode. The mass spectrometer condition was as follows: drying gas temperature, 225 °C; gas flow rate, 15.1 L/min; nebulizer pressure, 25 psi; mass range, 100–100 *m/z*; capillary voltage, 3000 V; skimmer voltage, 65 V; fragment voltage, 380 V; and collision energy 10, 20, 40, and 60 V.

As for QA derivatives, a high-performance liquid chromatography system (Water Alliance 2695, USA) equipped with a photodiode array detector was used for quantification of 3-*O*-caffeoylquinic acid (3-CQA) and 4-*O*-caffeoylquinic acid (4-CQA). A reversed-phase column, Luna^®^ C18 100 Å (4.6 mm × 150 mm, 5 µm), Phenomenex^®^ (USA) was used as the stationary phase. The mobile phase consisted of 0.1% formic acid in acetonitrile (A) and 0.1% formic acid in deionized water (B), which was programmed as follows: 0–15 min, gradient elution from 5A:95B to 20A:80B; then constantly held at 20A:80B for 2 min; 17–20 min; gradient running from 20A:80B to 5A:95B; then constantly held at 5A:95B for 4 min. The chromatographic conditions were set as follows: flow rate, 0.5 mL/min; injection volume, 10 µL; column temperature, 35 °C; and detection wavelength, 330 nm.

### Cellular antioxidation capacities of FDS extract on keratinocytes

#### Cell viability of human keratinocytes (HaCaT)

The HaCaT cells were seeded in a 96-well plate (20,000 cells per well) and cultured in 100-µL DMEM containing 10% FBS and 1% penicillin–streptomycin under 37 °C in a 5% CO_2_ incubator for 24 h. Next, the cells were pretreated with various concentrations of FDS extract (0–500 µg/mL), QA (0.10–6.60 µg/mL), 3-CQA (0.03–1.95 µg/mL), and 4-CQA (0.03–1.70 µg/mL) and incubated for the next 24 h. After that, the cells were challenged with or without 100-µM hydrogen peroxide (H_2_O_2_) in the incubator for 30 min. The cells were then incubated with an MTS reagent for 2 h. Finally, the absorbance was measured at the wavelength of 490 nm using a microplate reader (Spectramax, Molecular Devices, USA). The experiments were conducted in triplicate.

#### Determination of intracellular ROS

Intracellular ROS was measured using DCFH-DA, as previously described by Chansriniyom et al.^41^. The DCFH-DA was hydrolyzed to 2′,7′-dichlorodihydrofluorescein (DCFH) by cellular esterase. Then, DCFH reacts with intracellular ROS, leading to the formation of the fluorescent 2′,7′-dichlorofluorescein (DCF). HaCaT cells were seeded in a 96-well black plate containing 100-µL DMEM at a density of 20,000 cells per well for 24 h. Then, the cells were treated with the samples for 24 h. Before adding 10 µL of 10 µM DCFH-DA, the cell medium was removed, and the cells were rinsed with PBS. After 30 min of incubation, the cells were subsequently rinsed with PBS and incubated with or without 100-µM H_2_O_2_ for 30 min. DCF fluorescence was measured afterward using a microplate reader SpectraMax, Molecular Devices, USA) at excitation and emission wavelengths of 485 and 535 nm, respectively.

#### 3T3 neutral red uptake phototoxicity test

The phototoxicity of the FDS extract on 3T3 mouse fibroblast cells (ATCC^®^ CCL-92™; American Type Culture Collection, USA) was conducted via an in vitro 3T3 neutral red uptake, as described by the Organization for Economic Cooperation and Development (OECD) (TG 432) (OECD, 2019)^42^.

Briefly, the cells were seeded in 96-well plates at 10^4^ cells per well and cultured at 37 °C, 5% CO_2_ for 24 h. Then, the cells were pretreated with eight concentrations of FDS extract in 1X Hanks’Balanced Salt Solution (HBSS) in a range of 7.8–1000 µg/mL, and chlorpromazine hydrochloride (CPZ) was used as a positive control. After 60 min, the cells were irradiated with 5 J/cm^2^ of UVA, while the control plate was kept in the dark. After irradiation, the cells were washed twice with 1X HBSS and added a fresh medium to each well before being incubated for 18 h. Then, cell viability was determined using the neutral red uptake assay by measuring the absorbance at 540 nm. The half-maximal inhibitory concentration (IC_50_) of crude extract and CPZ in irradiated and non-UV-irradiated conditions, MPE, and photo-irritation-factor (PIF) were calculated using Phototox version 2.0 software, as per the OECD guidelines.

According to OECD TG 432, the endpoint levels of MPE and PIF can be predicted as (1) no phototoxicity, (2) equivocal phototoxicity, and (3) phototoxicity, with (1) MPE < 0.1 and PIF < 2, (2) MPE ≥ 0.1 and < 0.15 and PIF ≥ 2 and < 5, and (3) MPE ≥ 0.15 and PIF ≥ 5, respectively.

#### Skin irritation

The acute skin irritation caused by FDS extract was investigated on the reconstructed human epidermis (EpiDerm™, MatTek, USA) according to OECD (TG No. 439) (OECD, 2020)^43^. Briefly, the 3D skin model of the human epidermis was constructed by incubating the cells in the culture medium overnight at 37 °C with 5% CO_2_ and 90% relative humidity. Then, 30 µL of 830 mg/mL of the FDS extract was applied to the EpiDerm™ and incubated for an hour. The 5% SDS and sterile DPBS were used as positive and negative controls, respectively. After that, the 3D skin model was washed with PBS to remove the crude extract and further cultured in the culture medium for 18 h. The tissue viability was determined by MTT assay. MTT solution was incubated in the 3D skin model for 3 h; then, formazan was extracted, and measured the absorption at wavelengths of 570 and 650 nm. The extract solution is determined as a blank. Percentage tissue viability was calculated using the following formula:

$$\% \text{tissue} \; \text{viability}=100 \times \frac{(\text{OD}570 \; (\text{crude} \; \text{extract})-\text{OD}650 \; (\text{crude} \; \text{extract}))-((\text{OD}570 \; (\text{blank})-\text{OD}650 \; (\text{blank}))}{(\text{OD}570 \; (\text{negative} \; \text{control})-\text{OD}650 \; (\text{negative} \; \text{control}))-(\text{OD}570 \; (\text{blank})-\text{OD}650 \; (\text{blank}))}$$

### Statistical analyses

The experimental results were given as mean ± standard deviation (SD) of the three independent experiments. The data were analyzed via one-way ANOVA, followed by Tukey’s test (IBM, SPSS statistic version 22, USA). A significant difference is accepted when the *p*-value is less than 0.05.

## Conclusion

Phytochemicals in the aqueous extract of *F. dubia* sap were identified by LC–ESI–MS/QTOF-MS, revealing QA, syringoylquinic acid, 3-*O*-caffeoylquinic acid, 4-*O*-caffeoylquinic acid, and dimeric forms of caffeoylquinic acids. In the extract, the amount of QA, 3-*O*-caffeoylquinic acid, and 4-*O*-caffeoylquinic acid was 1.32 ± 0.15% w/w, 0.39 ± 0.01% w/w, and 0.34 ± 0.00% w/w, respectively. Furthermore, the radical scavenging activity of the *F. dubia* sap extract on keratinocytes was relatively correlated with 3-*O*-caffeoylquinic acid and 4-*O*-caffeoylquinic acid rather than QA. Furthermore, the *F. dubia* sap extract was determined to be safe for skin cells. In summary, the FDS extract is a promising extract that can be further developed as a new antioxidant ingredient for a skin healthcare product.

## Supplementary Information

Supplementary Information.
