# Supplementary material for: Tandem mass spectrometry of aqueous extract from Ficus dubia sap and its cell-based assessments for use as a skin antioxidant
Source: Sci Rep. 2021 Aug 19;11:16899. doi: 10.1038/s41598-021-96261-3 (PMC8377047; doi:10.1038/s41598-021-96261-3)
Supplement: Supplementary file 1 — Supplementary Information. [file 41598_2021_96261_MOESM1_ESM.doc]

**Supplementary materials**

**Tandem Mass Spectrometry of Aqueous Extract from *Ficus dubia* Sap and Its Cell-Based Assessments for Use as a Skin Antioxidant**

Chaisak Chansriniyom^1,2^, Rawiwan Nooin^3^, Nitra Nuengchamnong^4^, Ratjika Wongwanakul^3^, Nalinrat Petpiroon^3^, Wanwisa Srinuanchai^3^, Bhanumas Chantarasuwan^5^, Pornsiri Pitchakarn^6^, Piya Temviriyanukul^7^, Onanong Nuchuchua^3,*^

^1^Department of Pharmacognosy and Pharmaceutical Botany, Faculty of Pharmaceutical Sciences, Chulalongkorn University, Pathumwan, Bangkok, Thailand

^2^Natural products and Nanoparticles Research Unit, Chulalongkorn University, Bangkok, Thailand

^3^National Nanotechnology Center (NANOTEC), National Science and Technology Development Agency (NSTDA)

^4^Science Laboratory Center, Faculty of Science, Naresuan University, Phitsanulok 65000, Thailand

^5^National Science Museum, Technopolish, Klong 5, Klong Luang, Pathumthani, Thailand

^6^Department of Biochemistry, Faculty of Medicine, Chiang Mai University, Chiang Mai, Thailand

^7^Institute of Nutrition, Mahidol University, Nakhon Pathom, Thailand

* Corresponding author: Dr. Onanong Nuchuchua, Nano Agricultural Chemistry and Processing Research Team, National Nanotechnology Centre, National Science and Technology Development Agency, Tel.: +66 2 117 6792,
E-mail address: [onanong@nanotec.or.th](mailto:onanong@nanotec.or.th)


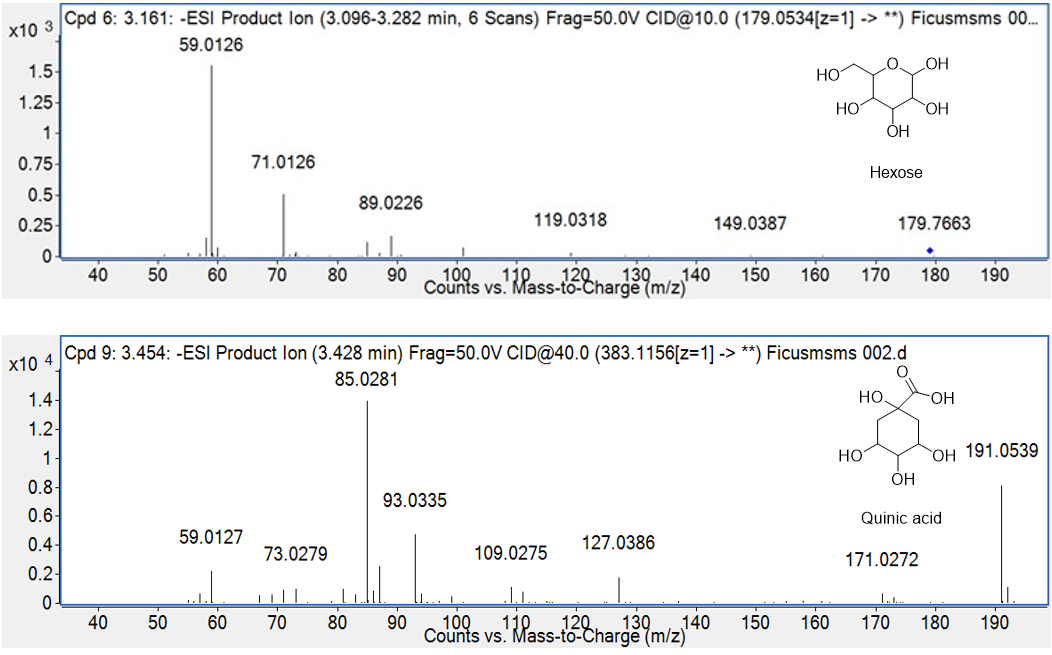


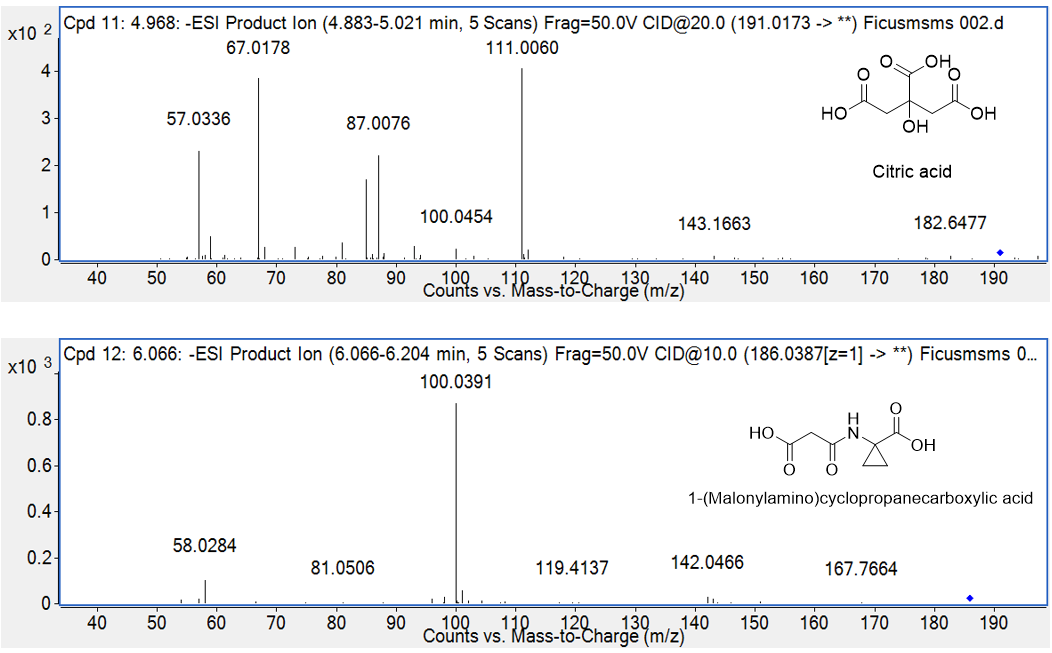


**Figure S1.** MS/MS data and structure of compounds found in *F. dubia* sap extract in the negative ionization mode.


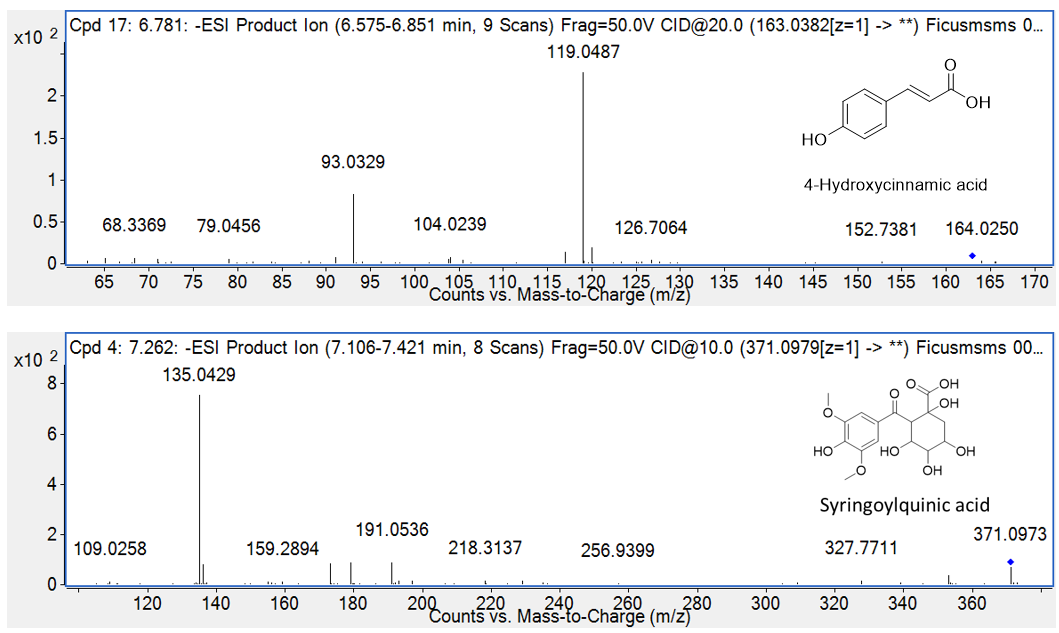


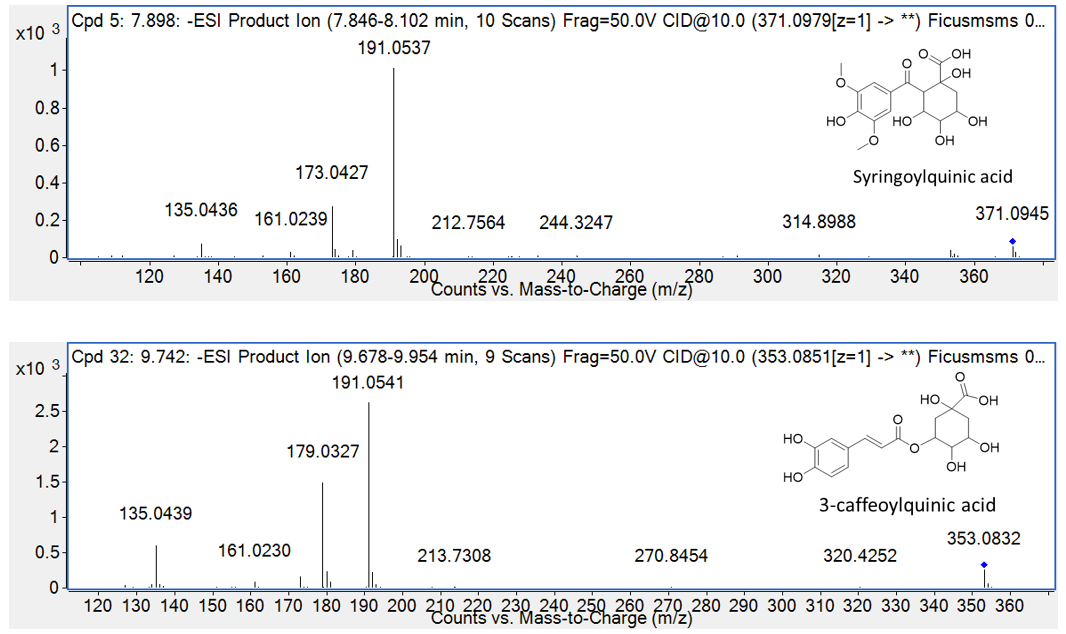


**Figure S1 (continued).** MS/MS data and structure of compounds found in *F. dubia* sap extract in the negative ionization mode.


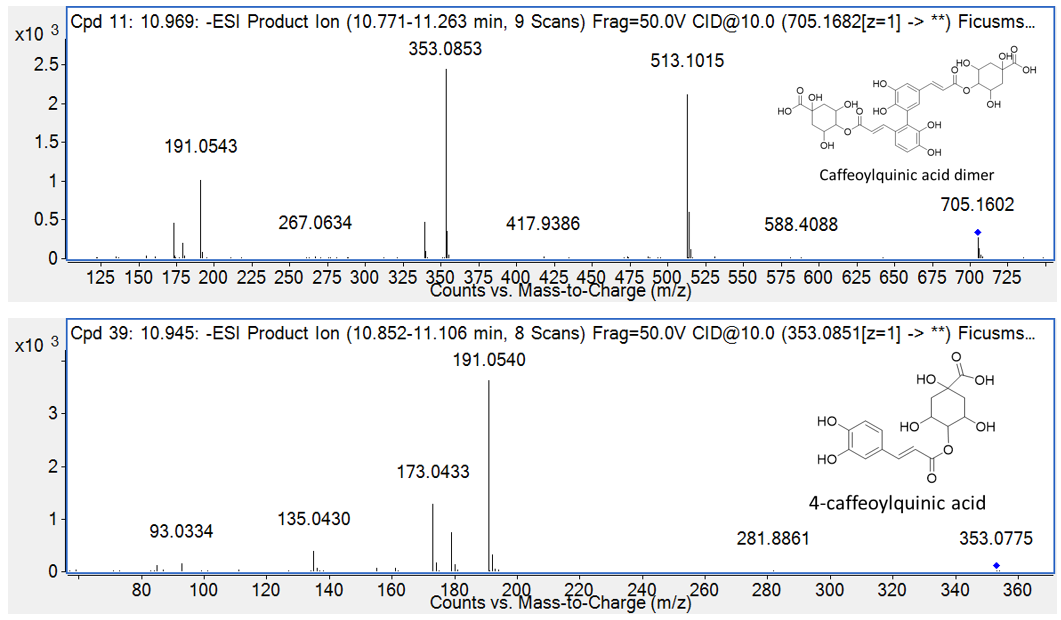


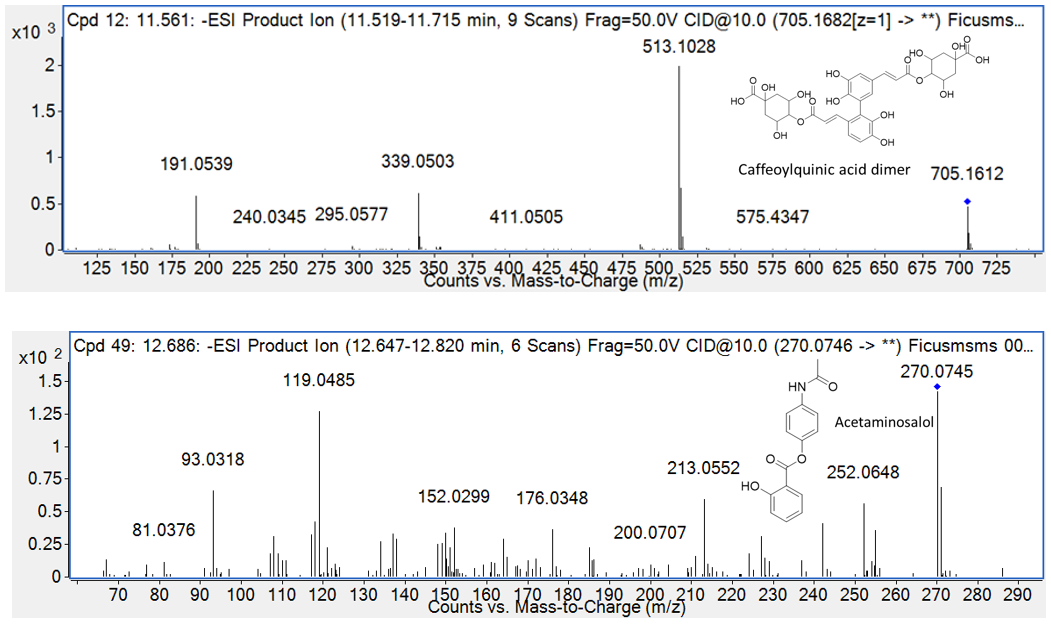


**Figure S1 (continued).** MS/MS data and structure of compounds found in *F. dubia* sap extract in the negative ionization mode.


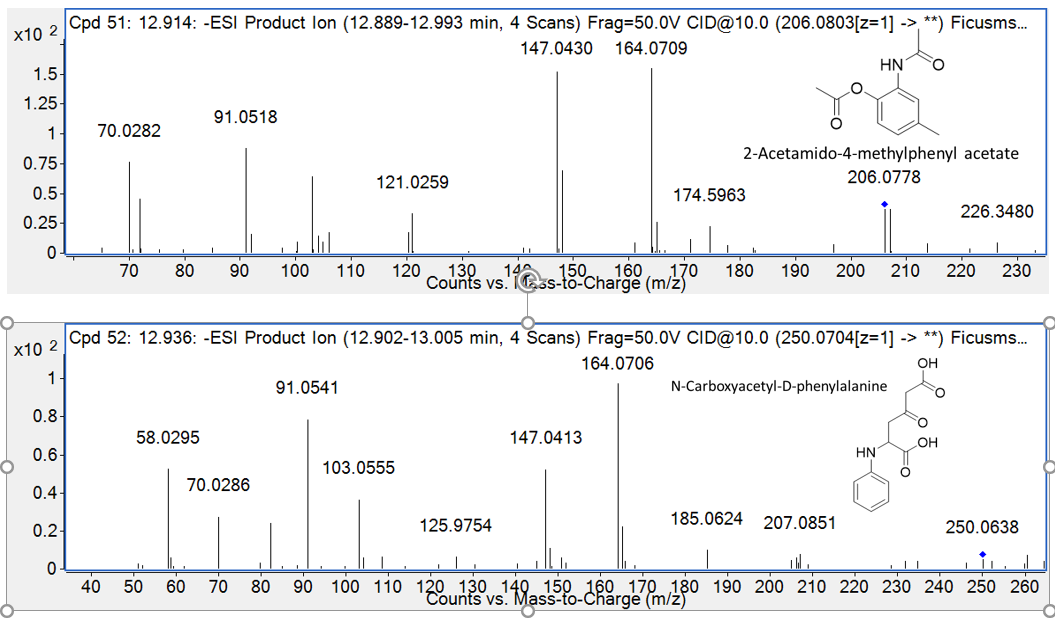


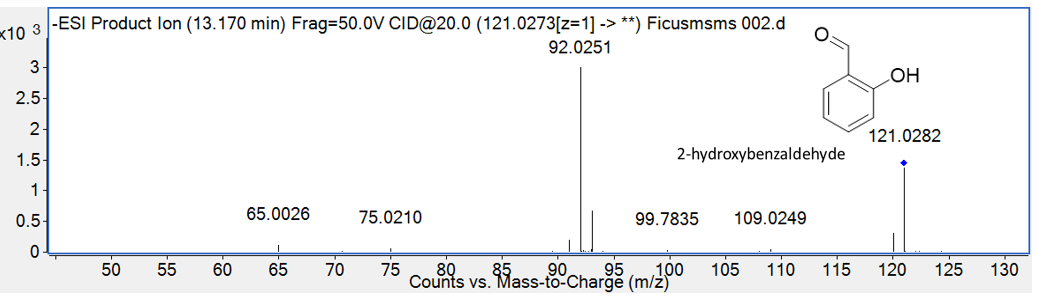


**Figure S1 (continued).** MS/MS data and structure of compounds found in *F. dubia* sap extract in the negative ionization mode.


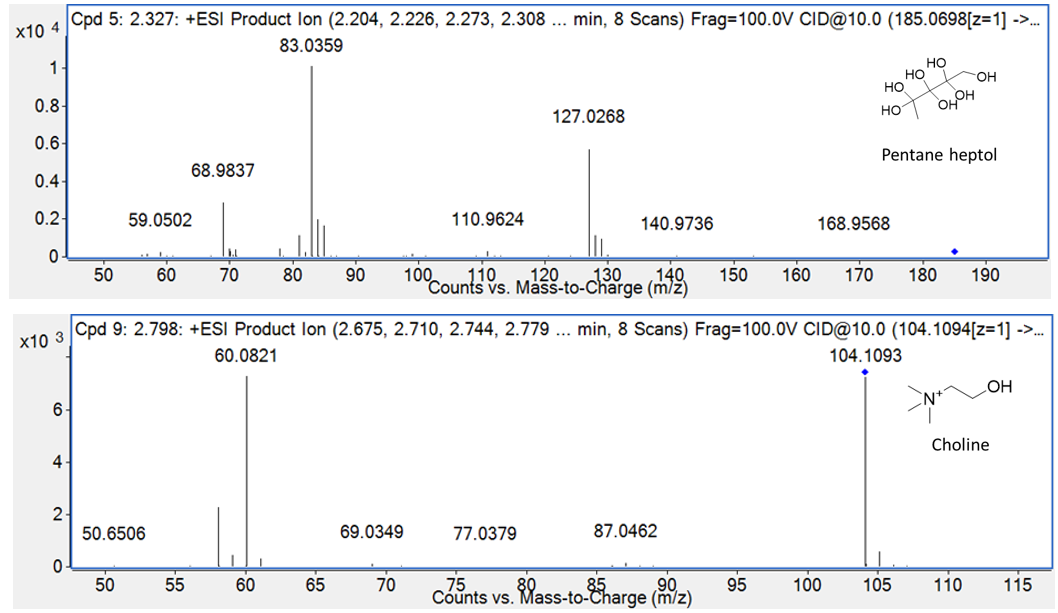


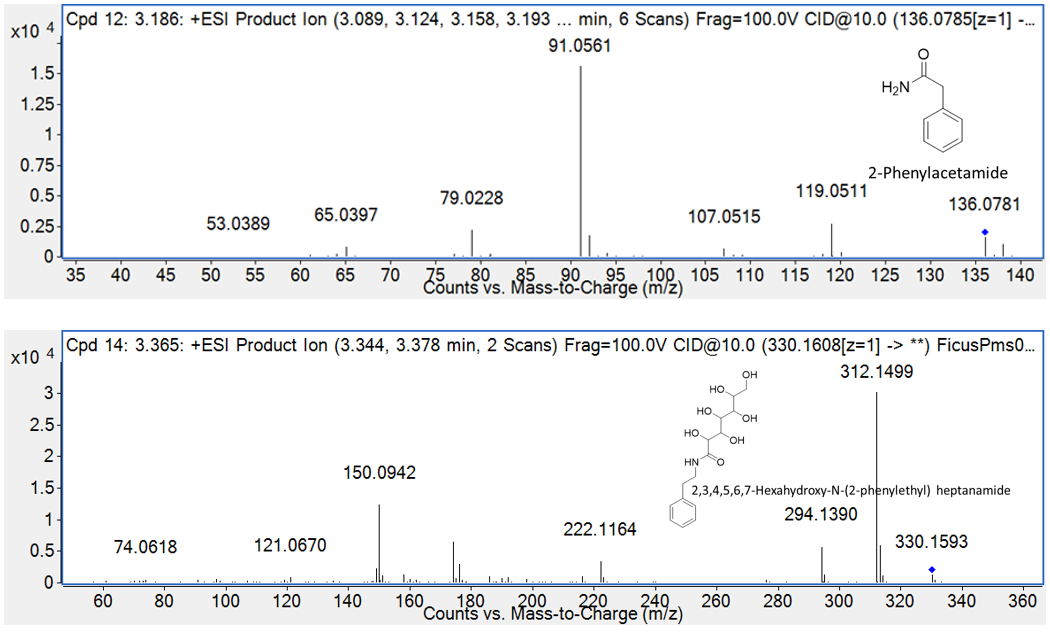


**Figure S2.** MS/MS data and structure of compounds found in *F. dubia* sap extract in the positive ionization mode.


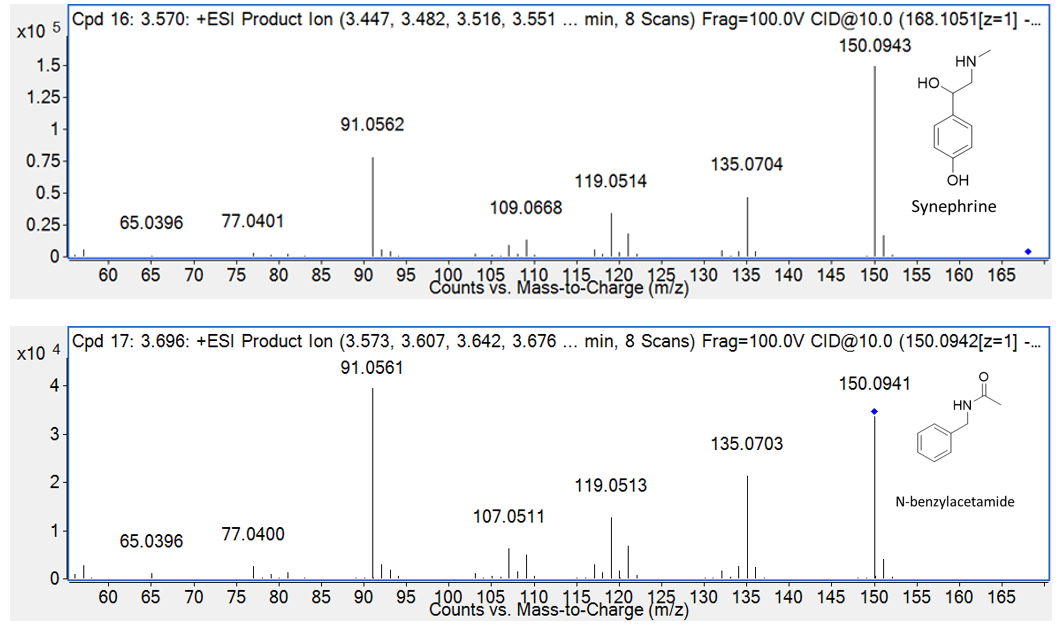


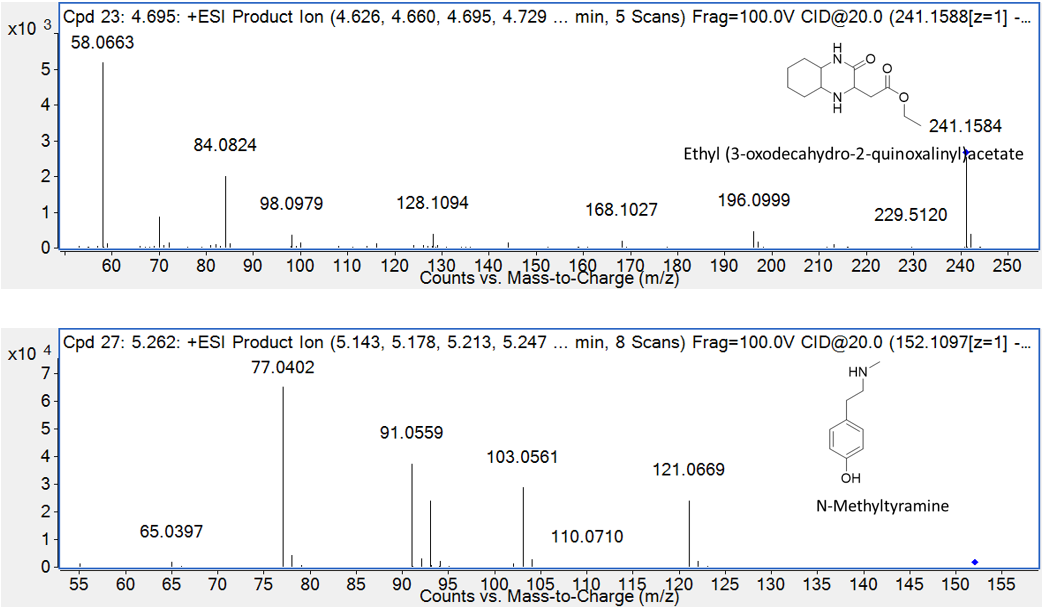


**Figure S2 (continued).** MS/MS data and structure of compounds found in *F. dubia* sap extract in the positive ionization mode.


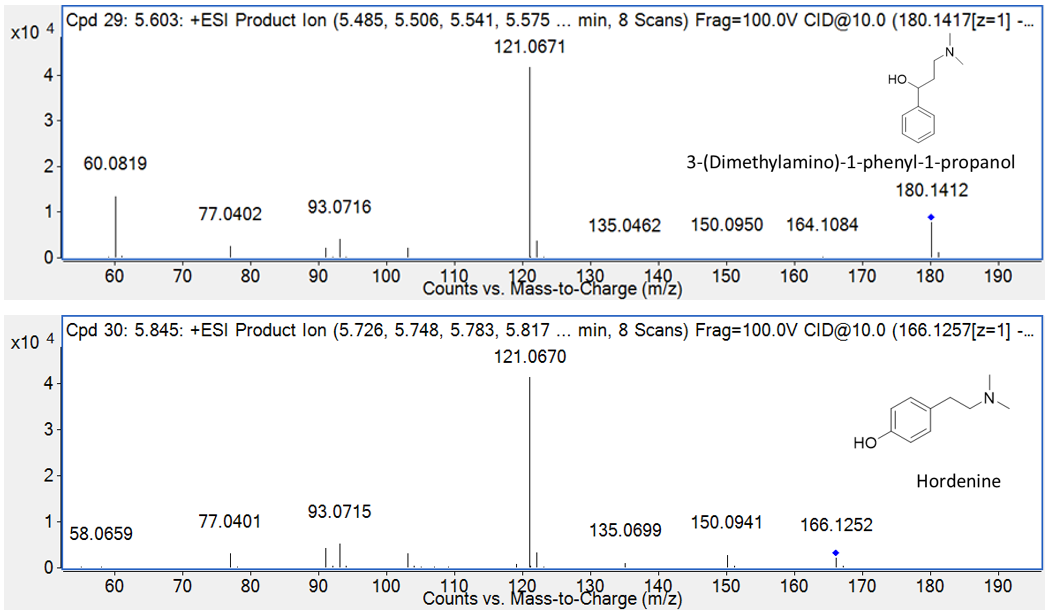


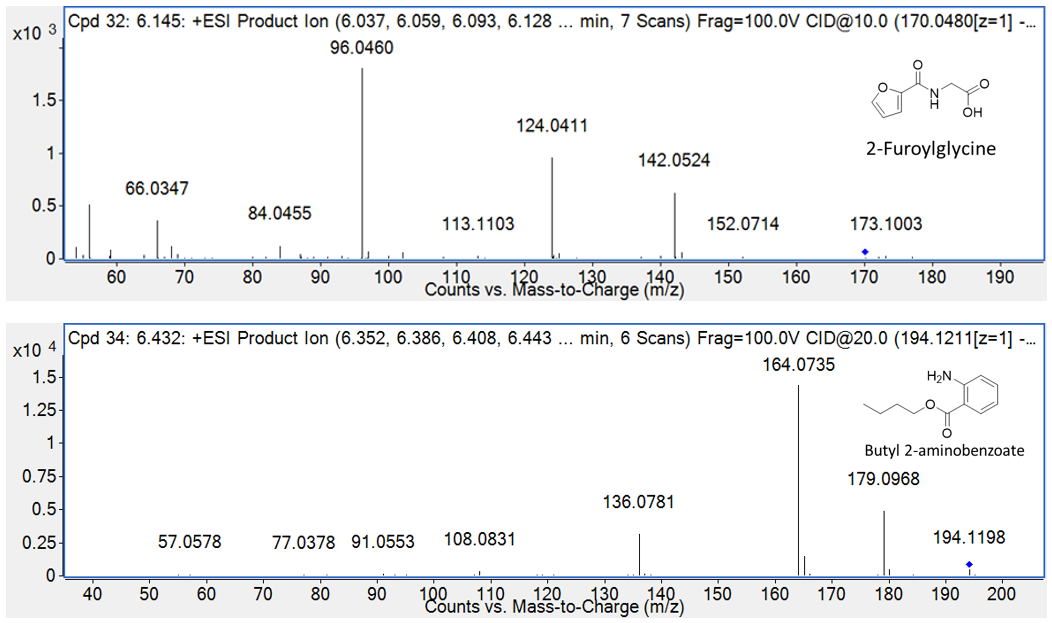


**Figure S2 (continued).** MS/MS data and structure of compounds found in *F. dubia* sap extract in the positive ionization mode.


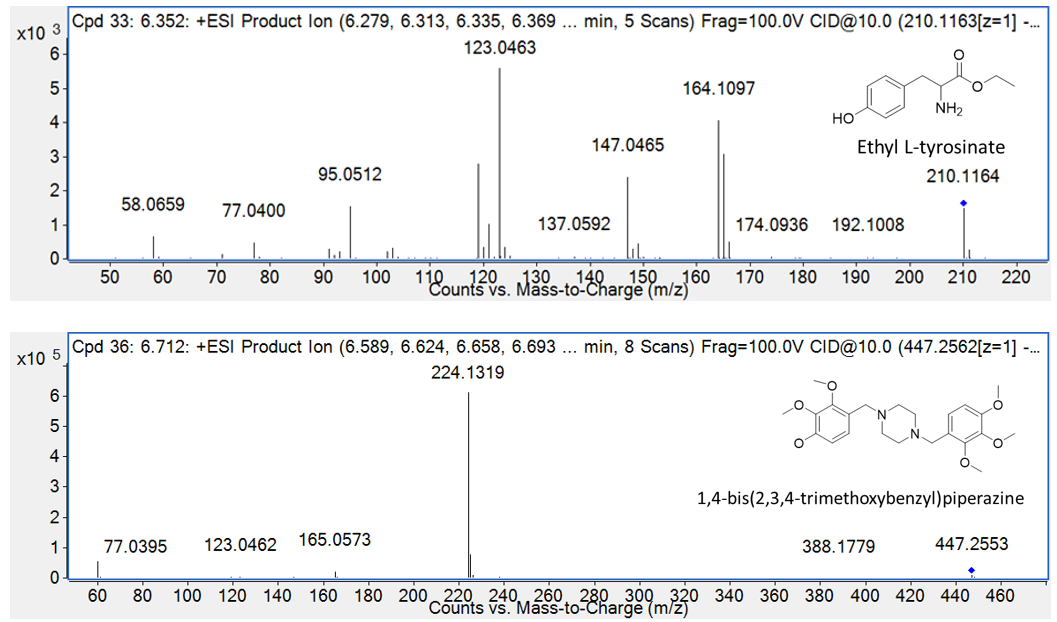


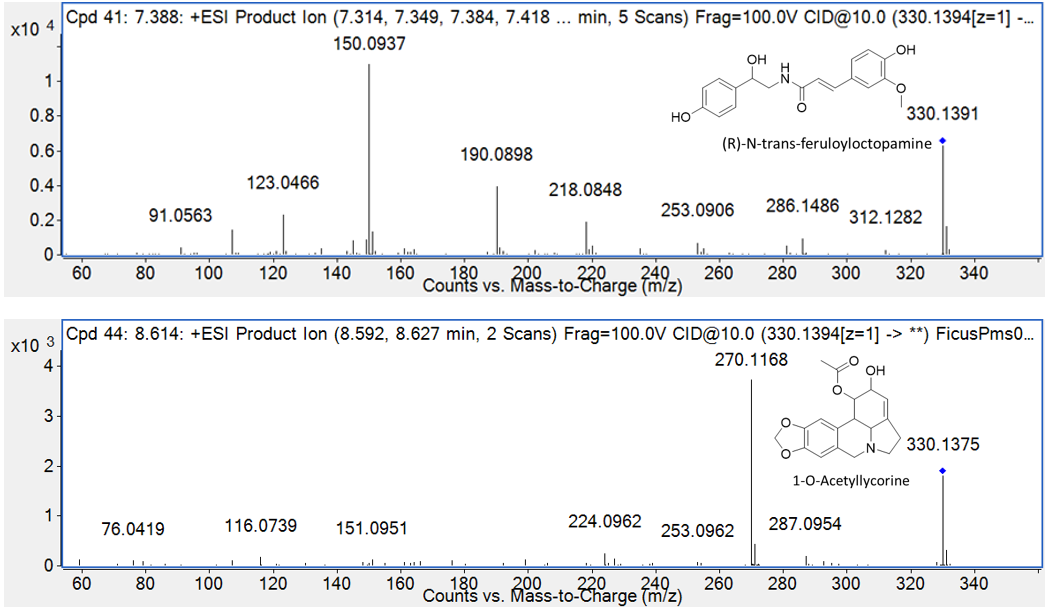


**Figure S2 (continued).** MS/MS data and structure of compounds found in *F. dubia* sap extract in the positive ionization mode.


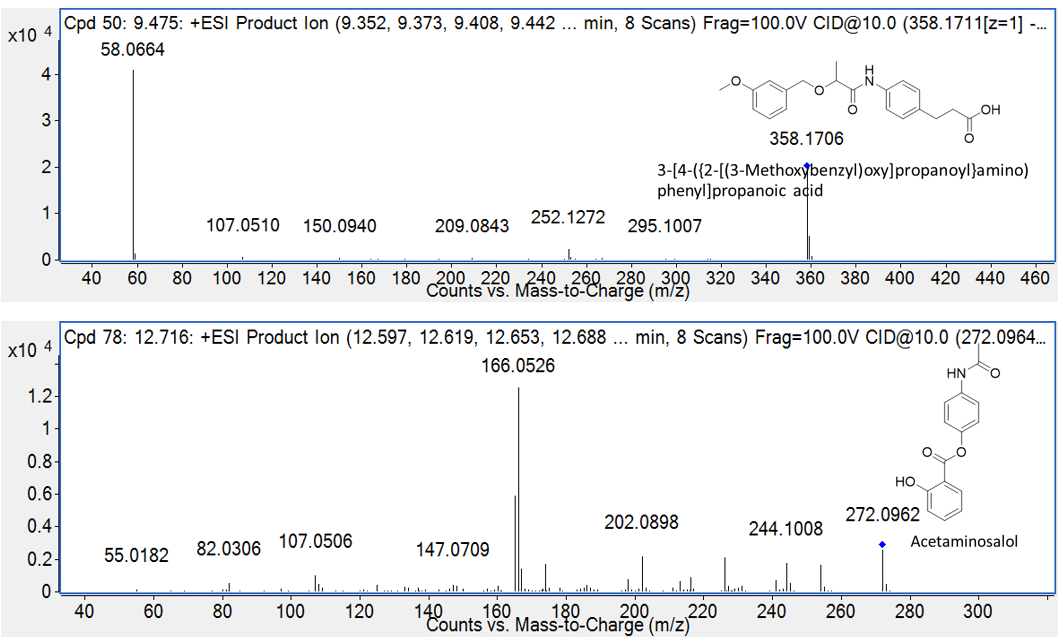


**Figure S2 (continued).** MS/MS data and structure of compounds found in *F. dubia* sap extract in the positive ionization mode.

**Table S1.** Analytical method parameters for the quantification of quinic acid (QA) and 3- and 4-*O*-caffeoylquinic acids (3-CQA and 4-CQA).

| **Standards** | **Linear range (ppm)** | **Linear regression equation** | **Correlation coefficient (R^2^)** | **LOD (ppm)** | **LOQ (ppm)** |
| --- | --- | --- | --- | --- | --- |
| QA | 0.0625-2.5 | Y=8666667x-82706.3 | 0.9980 | 0.01 | 0.04 |
| 3-CQA | 1-50 | y = 40766.7x – 48536.3 | 0.9949 | 1.02 | 3.09 |
| 4-CQA | 1-50 | y = 52517x – 27122.8 | 0.9989 | 1.04 | 4.10 |
